# Operation of a programmable microfluidic organic analyzer under microgravity conditions simulating space flight environments

**DOI:** 10.1038/s41526-023-00290-3

**Published:** 2023-06-08

**Authors:** Zachary Estlack, Matin Golozar, Anna L. Butterworth, Richard A. Mathies, Jungkyu Kim

**Affiliations:** 1 Department of Mechanical Engineering, University of Utah, Salt Lake City, UT 84112 USA; 2 Space Sciences Laboratory, University of California Berkeley, Berkeley, CA 94720 USA; 3 Biophysics Graduate Group and Chemistry Department, University of California Berkeley, Berkeley, CA 94720 USA

**Keywords:** Chemical engineering, Mechanical engineering, Biomedical engineering

## Abstract

A programmable microfluidic organic analyzer was developed for detecting life signatures beyond Earth and clinical monitoring of astronaut health. Extensive environmental tests, including various gravitational environments, are required to confirm the functionality of this analyzer and advance its overall Technology Readiness Level. This work examines how the programmable microfluidic analyzer performed under simulated Lunar, Martian, zero, and hypergravity conditions during a parabolic flight. We confirmed that the functionality of the programmable microfluidic analyzer was minimally affected by the significant changes in the gravitational field, thus paving the way for its use in a variety of space mission opportunities.

Microfluidics represents a major technological innovation for spaceflight due to its ability to manipulate micro- to nano-liter scale volumes of fluids and to perform highly sensitive chemical and biological analyses reliably with a small physical footprint. Microfluidic platforms are particularly appealing in the search for organic biosignatures of extra-terrestrial life. For example, the feasibility of gathering and microfluidically analyzing ice samples at Enceladus and Europa for biosignatures has been analyzed in detail^1–3^. Microfluidic bioanalysis systems may also be valuable for flight crew health monitoring. However, while powerful prototype microfluidic instruments have been developed and laboratory tested, the gravitational sensitivity of platforms that are compact, power-efficient, and reconfigurable for in-situ space exploration has not been examined^4–7^.

We have developed a microfluidic organic analyzer (MOA) system consisting of a programmable microvalve array (PMA) integrated directly with glass microchannels and a laser-induced fluorescence (LIF) detection system^8–14^. Previous MOA applications have focused on the performance of fluorescent labeling of amino acids, aldehydes, ketones, and carboxylic acids, followed by high-resolution capillary electrophoresis separation and nanomolar to picomolar detection of the labeled analytes^15–19^. More recently, the focus has been on the development of a Technology Readiness Level (TRL) 6 flight format instrument system that has a configuration and fabrication appropriate for space flight^20^. While preliminary thermal, vacuum, and vibration testing has been performed, zero gravity testing is more challenging in a terrestrial environment.

PMAs (described in more detail in Supplementary Fig. 1) are fully addressable pneumatic microfluidic valve arrays that can perform a nearly infinite variety of sample preparation and manipulation steps due to their programmability. Similar to a conventional logic circuit, pneumatically controlled microvalves can switch states, forming a processor^9,14^. When a vacuum is applied to a particular microvalve, a membrane in the microvalve deflects up to open the valve and pull liquid into its fluidic chamber. When the pneumatic state is switched to pressure, the liquid in the chamber is pushed out along a path determined by the states of the surrounding microvalves. If this opening and closing operation is performed in a sequence of connected microvalves, a net forward flow of fluid can be dispensed with high accuracy. With sequences similar to that shown in Supplementary Fig. 2, samples can be automatically labeled, incubated, manipulated, and delivered to the integrated capillary electrophoresis (µCE) chip for detection with the LIF system. We now desire to test a MOA, primarily its PMA and LIF systems, in a ZeroG parabolic flight to determine its performance sensitivity to reduced and hyper-gravity conditions.

We present here results from the first two flights in a series of five microgravity flights that will be conducted to evaluate the performance of MOA microfluidics in microgravity. Figure 1A shows the MOA system, including the PMA-µCE chip, manifold and LIF detection system, and sensor suite. The chip presented in Fig. 1B shows the rectilinear array of the microvalves acting as the fluidic processor as well as the other microvalves for controlling the fluidic path and reagent selection^21–23^. The chip is mounted on the manifold shown in Fig. 1C for pneumatic connections that control the PMA-µCE chip. The sensor suite described in Fig. 1C, incorporates control systems, environmental sensors, and experimental sensors for the flowrate and the LIF signal for use during the parabolic flight path (Supplementary Fig. 3). With this integrated MOA system, PMA performance was evaluated by first measuring the flowrate between two storage wells during multiple gravitational conditions. Second, the mixing and metering accuracy of the PMA was determined by diluting a fluorophore to specific concentrations and then using the LIF system to measure the concentration.

**Fig. 1:**
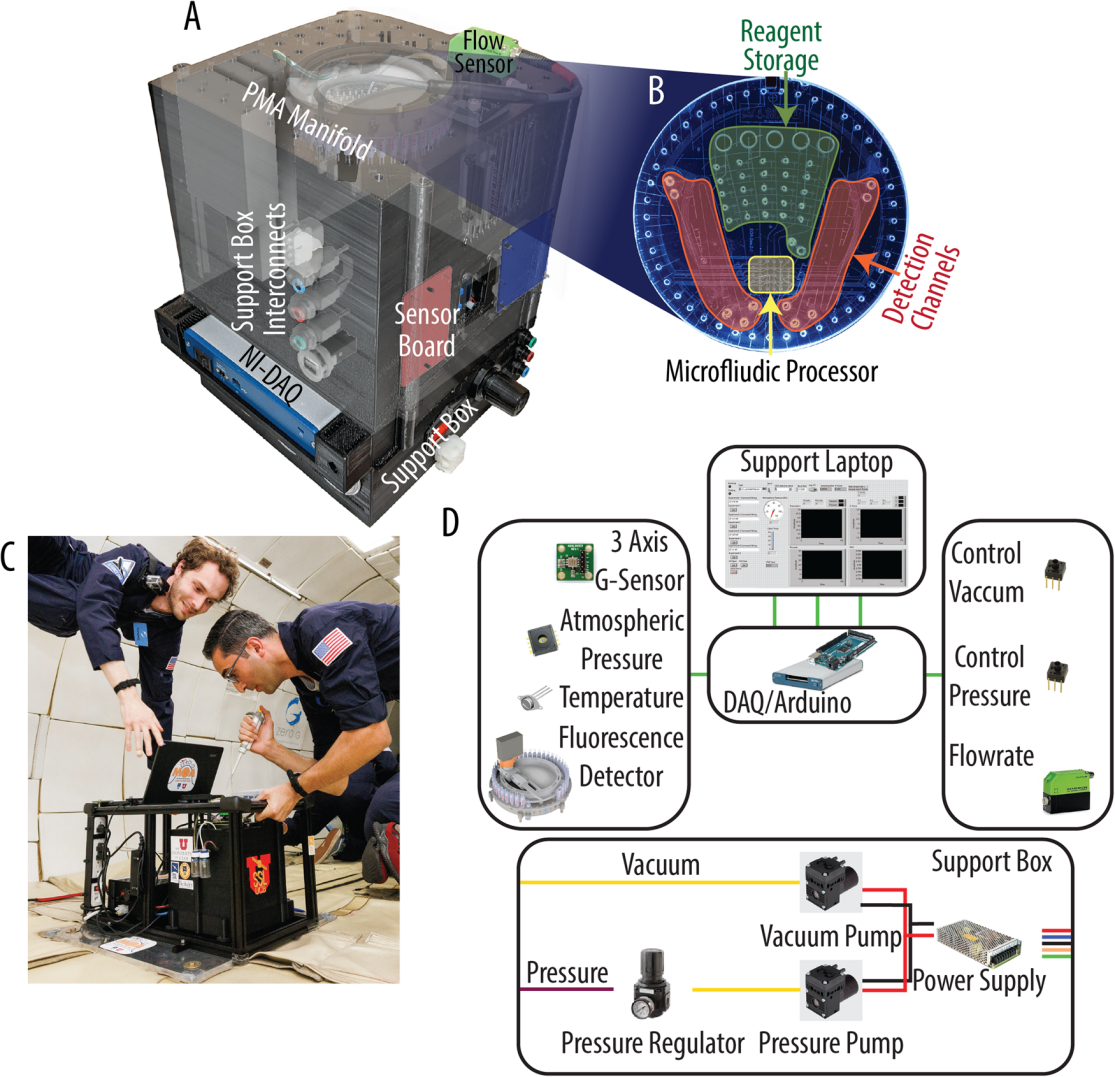
Apparatus used for flight testing of the microfluidic organic analyzer (MOA) consisting of the PMA-µCE chip, its operational hardware, and the sensor suite. **A** View of the main instrument indicating the internal component placement, including the sensor board, flowrate sensor, and NI DAQ used to obtain the environmental and experimental information. **B** PMA-µCE chip was tested with labeled sections: reagent storage for incubating and storing chemicals during analysis, the microfluidic processor that handles fluid delivery around the chip, and the detection channels where fluorescent dyes are pulled through LIF for measurement. **C** In-flight view of the testing apparatus being operated and monitored during microgravity exposure. Both individuals in the image have consented to their use. **D** System diagram for the sensors, control, and support equipment.

First, we assessed general operational parameters during flight to ensure that all testing environments were monitored and controlled as expected. Figure 2A shows that the atmospheric pressure dropped to 82 kPa as the plane climbed and pressurized, and there was an overall drop in temperature from 23.6 °C to 14.3 °C. The temperature change is not expected to have an impact on the overall performance of the MOA system, but it will affect the preparations for future flights that may involve temperature-sensitive reactions. The atmospheric pressure change can influence the pneumatic sources used by the PMA, lowering the vacuum level from −80 kPa at sea level to −64 kPa. The change in vacuum level does not significantly impact the valve dynamics as the 100 ms actuation time is much longer than the time required to deflect the membrane. However, there is a difference in displaced volume by the microvalve with −80 kPa displacing around 5% more volume than a microvalve operated at −64 kPa^13,21^. Figure 2B shows the pneumatic levels during one of the microvalve actuation sequences used during the flight. During actuation sequences, there is only a ~2.5 kPa change in vacuum, representing less than a 1% change in theoretical dispensed volume. Thus, we conclude that these changes in operational parameters will have minimal effect on the overall performance of the PMA for manipulating liquid samples when the pneumatic source pressures are matched.

**Fig. 2:**
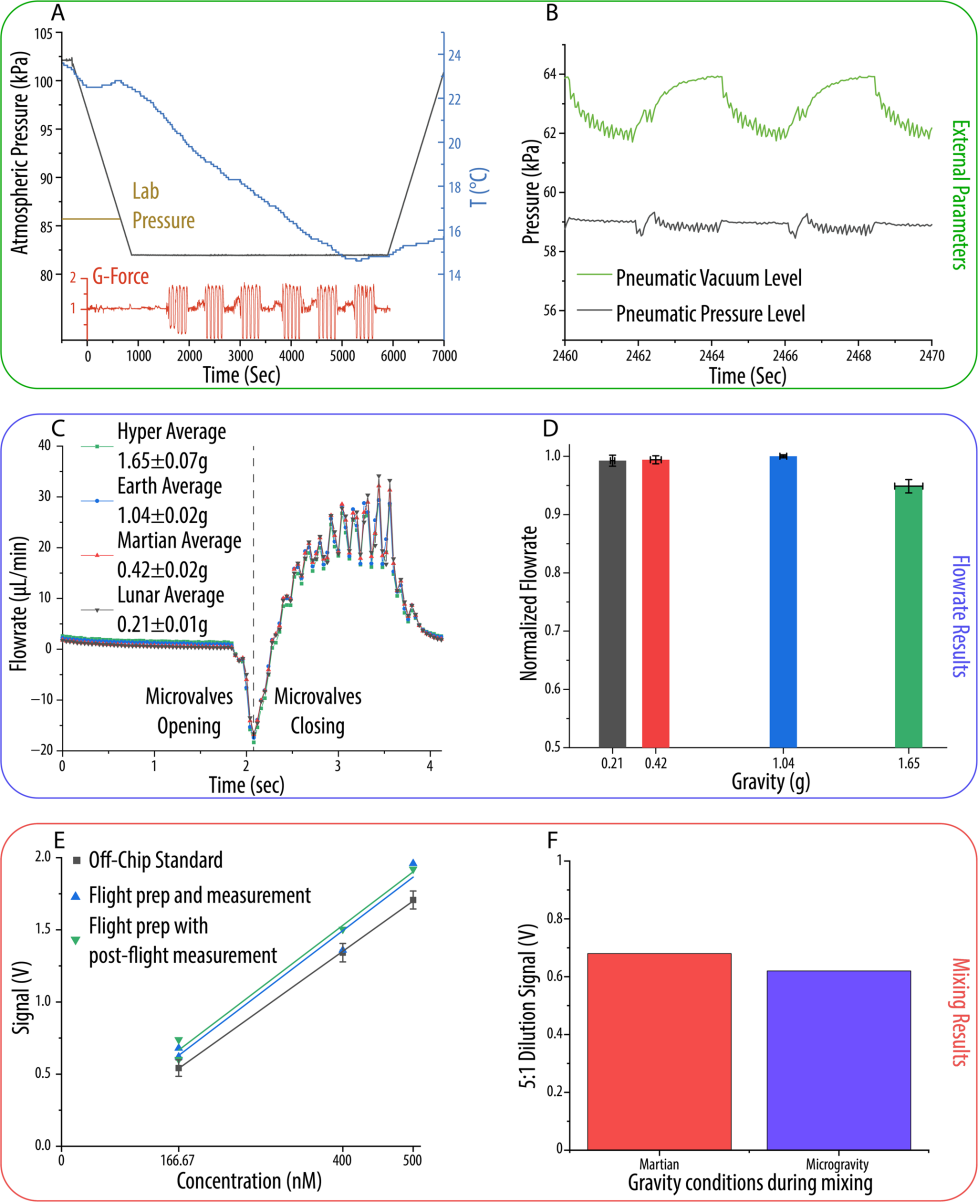
PMA environment and characterization during the flight. **A** G load, temperature, and atmospheric pressure changes during the flight. The atmospheric pressure was controlled by aircraft systems and is close to the pressure level in the testing laboratory at the University of Utah (1450 m; ~86 kPa on average). **B** Pneumatic levels measured during a pumping sequence. The fluctuation in the vacuum level occurs while valves are opening, and the pressure fluctuations occur during valve closing. **C** The flowrate profile for individual cycles at gravitational conditions ranging from 0.2 to 1.7 g. **D** The volume pumped per cycle under each of the different gravitational conditions (*n* = 8), normalized to 1-g gravity flow. **E** Plot of the resulting signal for the concentrations obtained through mixing. Each mixing sequence took place during alternating periods of micro- and hyper-gravity (between 0 and 1.7 g). **F** Results from dilution experiments. The lowest concentration was mixed twice, once under alternating micro- and hyper-gravity and again with Martian gravity, replacing the microgravity periods (between 0.42 and 1.7 g). Error bars on plots represent one standard deviation from the mean.

The flowrate measurement was then carried out during the Lunar, Martian, and hypergravity periods of the flight using the 4-s pumping sequence shown in Supplementary Fig. 2A. The sequence being performed during the microgravity flight is shown in Video 1. Figure 2C shows the range of flowrate profiles produced under different gravitational conditions after correcting for the hydrostatic pressure generated by the placement of the flowrate sensor 43 mm above the chip. Detailed calculations of the correction factors are included in the SI. The initial backflow when opening the outlet valve increased from −16.6 µL/min (Lunar) to −18.3 µL/min (hyper), and the peak flowrate in the middle of the pumping sequence decreased from 34.1 µL/min (Lunar) to 29.4 µL/min (hyper) with increased gravity. The Lunar case deviates 8% on average (±4.8 µL/min), and the Martian case deviates 6% on average (±2.6 µL/min) from the flowrate profile of average level flight during the closing portion of the sequence. These differences are within the typical sensor error range with a 25 Hz sampling rate. For the hypergravity case, the deviation is −1.7 µL/min at maximum (7% on average). However, the hypergravity profile is always lower than the level flight profile, in contrast to the Lunar and Martian cases that alternate between a higher and lower flowrate due to slight timing differences. This shows that our correction factor does not fully account for the variations between the hypergravity and the other three cases. Further investigation was carried out by estimating the volume dispensed per cycle for each case. Figure 2D shows the average volume per cycle pumped for each gravitational condition (*n* = 8). The Lunar and Martian cases pumped 99.25 ± 0.1% and 99.4 ± 0.6% of the level flight average, respectively. This level of variation is common over longer period flowrate testing^22^ and corresponds to ~3 nL of difference overall volume per cycle. However, the volume pumped per cycle during hypergravity was 94.87 ± 1.1% of the average value during level flight. This difference is partially due to the dynamic change of the hydrostatic backpressure while the plane ascended at the beginning of each series of parabolas. The height of the flow sensor was measured perpendicular to the floor of the aircraft; hence, during acceleration towards the top of the parabola, the effective hydrostatic height difference increased (Supplementary Fig. 4), reducing the volume pumped per cycle more than during level flight. During the hypergravity period, the gravitational vector is approximately 5° from vertical which can influence the volume per cycle during hypergravity, changing the volume per cycle to 94.95 ± 1.1% of the level flight amount. Taking into account the potential error from the relatively modest sample rate (25 Hz) of the flowrate sensor and the interruptions of cycles due to gravitational changes, we conclude that the gravitational environment has little impact on the performance of the PMA pumping capability.

With our precise volume control capability under varied gravitational conditions, automated dilutions were conducted to assess the metering and mixing performance of the PMA in preparation for future biomarker assay testing. The dilution sequence for this experiment took place in three stages (Supplementary Fig. 2B), and a recording of the sequence during flight is shown in Video 2. The first two stages involved transferring a buffer (30 mM sodium borate, pH 9.2) and a fluorophore (1 µM resorufin) to a storage well in the desired ratios (5:1, 3:2, 1:1), and the final stage involved loading 10 µL of the diluted fluorophore into the integrated detection channel and pulling it past the LIF detector using vacuum. Figure 2E shows the results for manual off-chip mixing compared with mixing samples by the PMA in-flight. The resulting measurements correspond to a linear R-Square value of 0.987 for the dilutions mixed and measured during flight. Additionally, the manually prepared standard had a slope of 3.48 $\frac{{mV}}{{\mu M}}$, while the dilutions performed on-chip during flight were 3.71$\frac{{mV}}{{\mu M}}$ regardless of measurement conditions. There is a difference of 0.09 V between the y-intercepts of the on-chip and off-chip dilutions due to differences in microfluidic resistance and the varied flowrate generated by different gravity conditions. Incorporating these aspects into the calculation of the expected concentration of the dilutions results in a slope of 3.50 $\frac{{mV}}{{\mu M}}$ and a y-intercept 0.12 V below that of the standard (Supplementary Fig. 5), most likely due to the dead volume of buffer present on the chip during the initial stages of the mixing process. In practice, the fluidic resistance and dead volume can be accounted for during sequence formulation to obtain a desired concentration at the end of a process. Furthermore, Fig. 2F shows that the signal obtained from a particular dilution sequence showed little variation (~17 nM or ~10%) when performed under microgravity or Martian gravity. This small variation in fluorescence is acceptable, matches the variation seen in the manually prepared standard, and would likely decrease with the opportunity for more statistical testing.

The results from these first two flights are an important step in the process of enhancing the TRL of the MOA platform. More generally, the successful performance of microfluidics in microgravity provides justification for their inclusion in spaceflight missions. We found that pumping performance remains constant as gravity decreases, but there is some degradation in performance with increased gravity up to the 2 g level tested. This degradation is likely due to measurement error and minor hydrostatic effects induced by the flowrate measuring equipment location rather than the function of the PMA chip itself. The mixing, metering, and detection capabilities of the MOA platform were unaffected by gravitational conditions, showing strong linear trends and a concentration difference of ~10% between samples prepared under different gravitational conditions. This variation is comparable to the error observed with manual preparation at all concentrations (~16 nM). The combination of these two results shows the potential for MOA devices to be used in extra-terrestrial chemical and biochemical analysis applications. The insensitivity of microfluidics to the gravitational field also justifies the initial development of microfluidic instruments in laboratory settings for eventual space deployment.

Importantly, lessons learned during this first flight campaign will be impactful on future microgravity flights and experiments through equipment and protocol improvements. Upcoming microgravity flights are planned to test two different use cases for the MOA in space exploration. The primary use case will be investigated by performing capillary electrophoresis during microgravity/hypergravity cycles, an analog for analysis of samples in-situ for the search for extra-terrestrial life^24^. Second, the use of the MOA system as a crew health monitoring platform will be demonstrated by performing mock clinical assays of relevant astronaut biomarkers in microgravity. The MOA platform is being updated based on the lessons learned from the flight, including the implementation of LED indicators to monitor experimental status, the addition of high voltage circuits for µCE, and the modification of the current layout to improve accessibility so that it can serve as a robust and reliable platform for future microgravity experiments and additional fieldwork. The outcomes of this and our future flights will demonstrate the capability of MOA for a variety of space missions, including crew health monitoring platforms as well as the search for signs of extra-terrestrial life.

## Methods

### Programmable microvalve array (PMA) fabrication^21–23^

PMA fabrication followed conventional soft lithography methods to generate fluidic and pneumatic polydimethylsiloxane (PDMS) layers. These were bonded together through oxygen plasma treatment after all necessary access ports were punched through the pneumatic layer. After bonding, all fluidic access ports were punched, and the bonded layers were cleaned in preparation for Trichloro(1H,1H,2H,2H-perfluorooctyl)silane treatment of the microvalves^22^. This is necessary due to the normally closed nature of the microvalves causing the gate structure to contact the surface of the µCE chip during bonding. Thus, a microcontact printing-based method is used to prevent bonding of the gates while allowing normal bonding to occur throughout the rest of the device.

### Experimental platform fabrication

To obtain relevant experimental results as well as safely operate the PMA during the microgravity flight, an experimental platform consisting of two enclosures, a support frame, and a base plate were fabricated. The first enclosure was designed to house the electrical and pneumatic supplies, keeping them away from the sensitive experimental apparatus and the second enclosure housed the PMA in its manifold, a LIF based detection system, and sensors for environmental parameters like gravity and temperature, as well as operational parameters like pneumatic levels and a flowrate sensor. The support frame was included to provide a location for mounting the boxes as well as the control laptop used to run experiments and obtain data. In addition, it provided the necessary structural support to safely operate the platform on an aircraft. Last, the baseplate was fabricated to mount the overall system to the frame of the aircraft.

### Organization of flights and experiments

The general organization of the microgravity flights is to break them up into six sets of five parabolas each. Each parabola generates 15 to 20 s of microgravity and there is a period of hypergravity in between each microgravity period. In addition, the first two parabolas were Martian gravity and the next three were Lunar gravity. The remaining flights were all microgravity. The results of these experiments come from two separate flights. The first flight was scrubbed after the first set of five parabolas allowing for reorganization for the second full flight to gain different results than originally expected. The first flight consisted of a flowrate test in which the microfluidic processing unit (MPU) pumped fluid from a reservoir to the flowrate sensor for the entire five parabola set giving volume per cycle and instantaneous flowrate data for Lunar, Martian, and hypergravity conditions. Coupled with a test at level flight, flowrate data for four different gravitational conditions was obtained. The second flight focused on characterization of the mixing capabilities of the PMA. Dilutions of Resorufin were completed by the MPU before being sent through the detection channel autonomously. A 1:5 dilution (Resorufin to water) was done at Lunar and Martian gravity and repeated at microgravity to determine gravitational effects. Dilutions of 2:3 and 1:1 were also completed in microgravity conditions. The dilution process was started at the start of the first microgravity parabola and ran continuously through all five parabolas in that set including the hypergravity periods.

### PMA actuation sequences

For the mixing experiment, the PMA moves buffer from one storage well to another well that is connected to the flowmeter (Supplementary Fig. 2A). The mixing experiment was operated in three parts. In Supplementary Fig. 2B1, the PMA pulls in buffer from the buffer inlet reservoir and delivers it to one of wells for the diluted samples depending on desired dilution. During Supplementary Fig. 2B2, the PMA delivers resorufin (red wells) to the same dilution storage well. These two steps are repeated at the desired ratio (5:1, 3:2, 1:1) to dilute the stock 1 µM resorufin. Last, in Supplementary Fig. 2B3 the PMA delivers ~10 µL of the diluted sample to a well attached to the detection channel where a vacuum pulls it over the LIF detector through the detection channel.

### Flowrate measurement processing

First, the flowrate data were corrected using the correction factor found using the process given in the Supplementary Methods section. Next, each flowrate profile was integrated numerically using a MATLAB script with the resulting value corresponding to the volume dispensed during that cycle. The midpoint of the sequence was also identified using MATLAB and flowrate profiles from matching gravitational conditions were averaged point by point to obtain the averaged flowrate profile shown in Fig. 2C.

### Reporting summary

Further information on research design is available in the Nature Research Reporting Summary linked to this article.

## Supplementary information

Supplementary Materials

Video 1-Flowrate Test Clip

Video 2-Mixing Experiment Clip

Reporting Summary

## Data Availability

The data collected during this study are available from the corresponding author upon reasonable request.

## Code availability

All scripts and computer code necessary to collect and analyze the data collected during this study is available from the corresponding author upon reasonable request.
