# Supplementary material for: Operation of a programmable microfluidic organic analyzer under microgravity conditions simulating space flight environments
Source: NPJ Microgravity. 2023 Jun 8;9:41. doi: 10.1038/s41526-023-00290-3 (PMC10247770; doi:10.1038/s41526-023-00290-3)
Supplement: Supplementary file 1 — Supplementary Materials [file 41526_2023_290_MOESM1_ESM.pdf]

SUPPLEMENTAL INFORMATION

Title: Operation of a programmable microfluidic organic analyzer under microgravity conditions simulating space flight environments

*Zachary Estlack[1], Matin Golozar[2], Anna L. Butterworth[3], Richard A. Mathies[2,3], and Jungkyu Kim[1]**

[1]Department of Mechanical Engineering, University of Utah, Salt Lake City, UT 84112
[2]Biophysics Graduate Group and Chemistry Department, University of California Berkeley, Berkeley, CA 94720
[3]Space Sciences Laboratory, University of California Berkeley, Berkeley, CA 94720

Due to the subject matter no ethical approval was sought for this work.

Supplementary Methods

Gravitational correction and volume per cycle calculation

The center of the flowrate sensor inlet was 43.5mm above the fluidic layer of the PMA during the microgravity flights. Using this height difference and assuming a constant gravitational case, a back pressure during each of the gravitational conditions can be determined with Supplemental Equation 1.

$$(1)\ \Delta P = \rho * g * G_f * h$$

Where ρ is density of water, g is gravitational acceleration, G_f is the g-force factor $(0.2, 0.4, 1, 1.7)$ for the Lunar, Martian, Earth, and hypergravity cases, respectively, and h is the height difference. Additionally, the microfluidic resistance of the flow path through the PMA as well as the connecting tubing to the flowrate sensor can be calculated using Supplemental Equation 2 & Supplemental Equation 3.

$$(2)\ R_{rec} = \frac{12\mu L}{1 - 0.63\left(\frac{h}{w}\right)} * \frac{1}{h^3 w}$$

$$(3)\ R_{cir} = \frac{8}{\pi}\mu L * \frac{1}{r^4}$$

Where R_{rec} is the fluidic resistance in the rectangular channels of the PMA, μ is the dynamic viscosity, L is the fluidic path length, h is the height of the fluidic channel, w is the width of the fluidic channel, R_{cir} is the fluidic resistance of the tubing connecting to the flow meter, and r is the radius of the tubing. The equivalent fluidic resistance, R_{eq}, for a path is calculated the same as conventional electric circuits. Using a circuit analog for microfluidic flow, Supplemental Equation 4, the backflow generated by the hydrostatic pressure difference can be calculated.

$$(4)\ Q = \frac{\Delta P}{R_{eq}}$$

This backflow was then added to the measured flowrate during the experiment to correct for the backpressure and then the overall flow profile was integrated to determine the volume per cycle for each gravitational case.

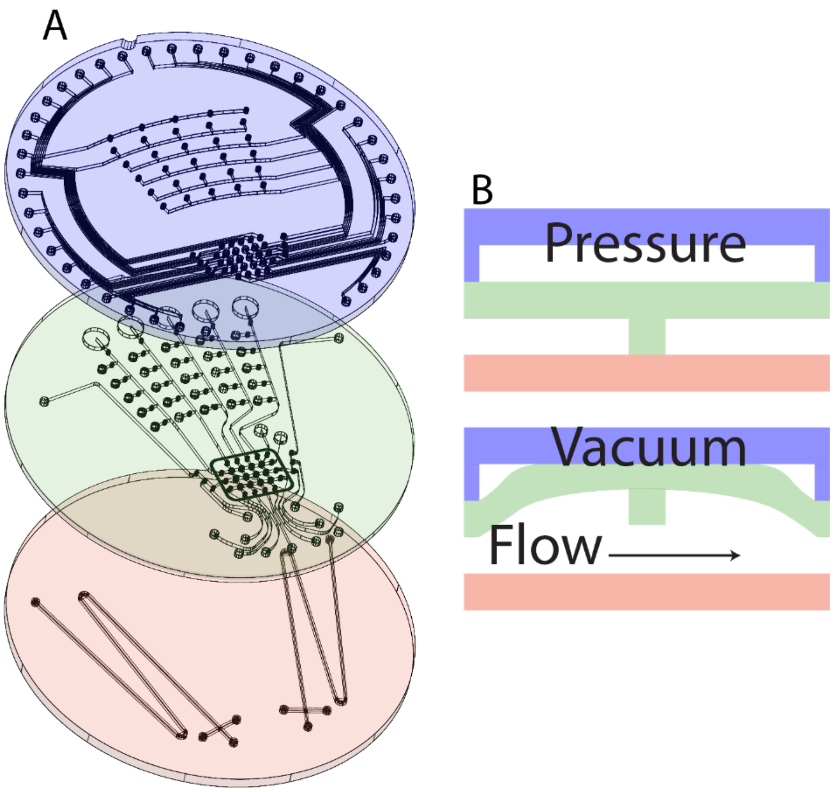

Supplementary Figure 1. PMA-µCE chip assembly. (A) Exploded view of the layers of the PMA-µCE chip (fluidic layer (green), the pneumatic layer (blue) and µCE chip (red)). The top and middle layers are fabricated using PDMS and conventional soft lithography, and the bottom layer is glass and fabricated with glass etching and bonding. The top layer is 4.5mm thick with 80 µm thick pneumatic channels that direct the applied pressure or vacuum to the desired microvalve. The middle layer is 250 µm thick with 50 µm high and 250 µm wide fluidic channels with the microvalve membranes and gates. These react to the pressure or vacuum states to open or close each microvalve on the chip. The fluidic channels are 250 µm wide to limit the impact of fluidic resistance and allow for faster operation. The bottom layer is a µCE chip that is used as a detection channel in these experiments. The µCE channel is 30 µm high and 110 µm wide. The three layers are integrated through oxygen plasma exposure bonding, with precise alignment ensuring the correct interfacing of the three layers. (B) The cross section of the fluidic layer (green) and how it interacts with the pneumatic layer (blue) and µCE chip (red) during both pressure and vacuum. The PMA can produce up to 850 nL per cycle of net forward flow under normal use and conditions with this simple pumping setup.

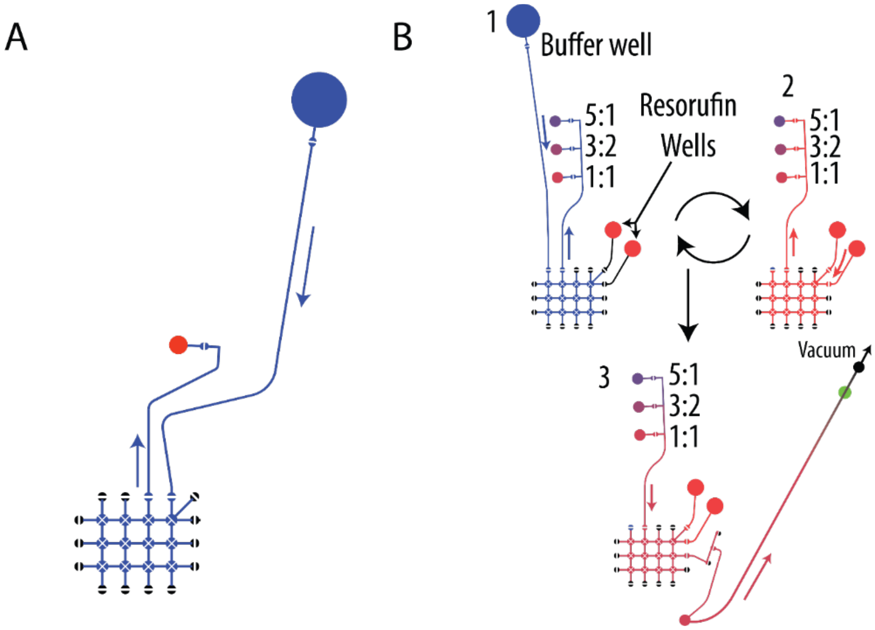

Supplementary Figure 2. Flow paths for each of the experiments performed during the microgravity flight. (A) The sequence used during the flowrate experiment, pulling from one storage well and pumping to another that is connected to the flowrate sensor. (B) Sequence used to test different mixing ratios, split into the three main components: buffer delivery, resorufin delivery, and loading and detection of diluted sample.

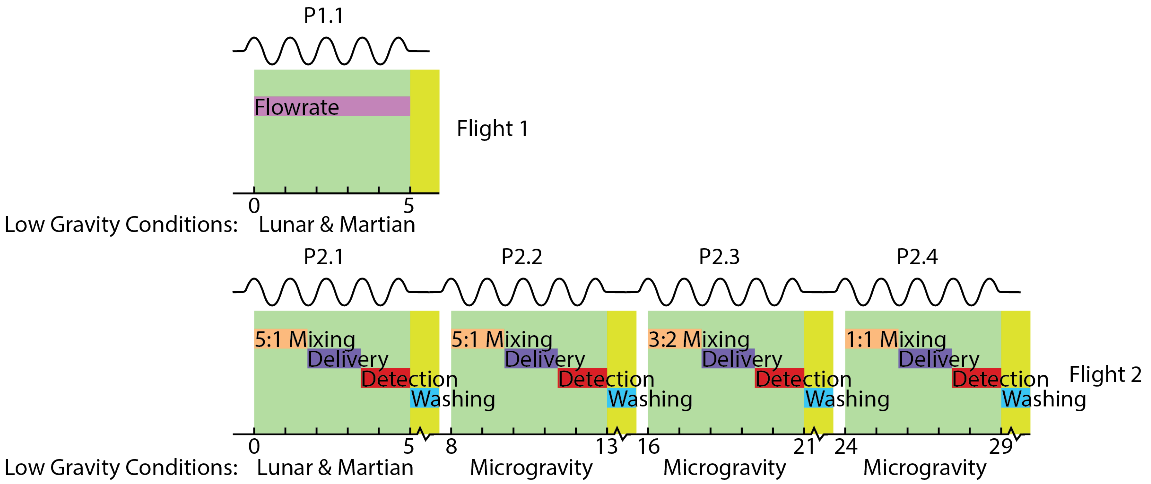

Supplementary Figure 3. Schedule of in-flight testing. The green regions designate periods of alternating low gravity and hyper gravity. Yellow is a period (~3 min) of level flight in between periods of low gravity parabolas. The line at the top of each flight designates a rough flight profile and the gravitational conditions are designated at the bottom. (Top) Flight 1 consisted of flowrate testing under Lunar & Martian gravity only as a mechanical issue with the aircraft forced the flight to end early. This allowed for changes to the experimental plan for the repeat flight. (Bottom) Flight 2 focused on mixing characterization, the chip sequentially mixed, delivered, and detected the designated mixtures of borate buffer and resorufin. In between P2.1 and P2.2 and after P2.4 the mixture volume remaining in the respective storage wells was removed for subsequent ground measurement verification. The two remaining parabola sets were designated as backup sets that were used for functional testing not relevant to this manuscript.

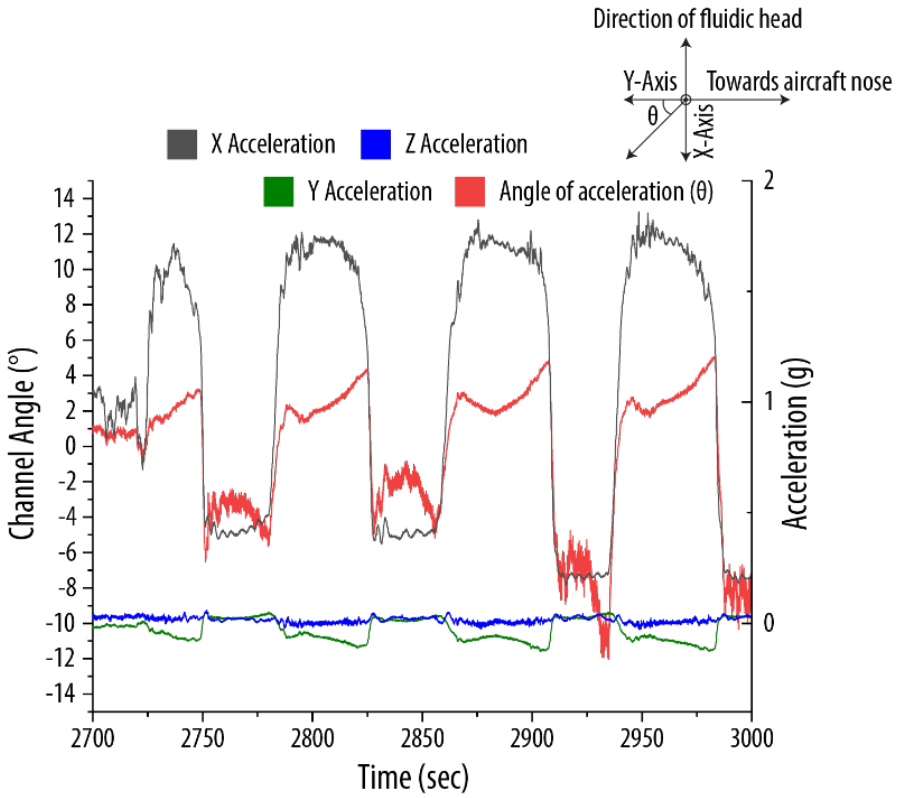

Supplementary Figure 4. Plot of the acceleration during Martian and Lunar parabolas as well as the resultant acceleration.

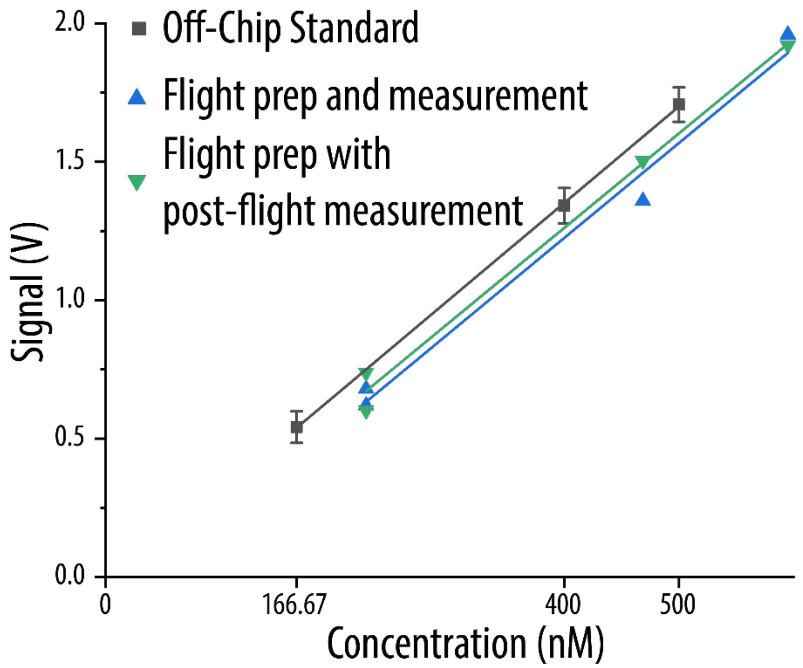

Supplementary Figure 5. Results of the mixing experiment after correcting for the flow differences brought about by fluidic resistance. The shift of the flight prepared samples to the right is caused by the fluidic resistance of the chip causing differing volumes to be delivered at each part of the experiment, changing the ratios from the intended values. The slope of the off-chip standard and flight prepared samples differs by only $0.02\frac{mV}{\mu M}$ and the y-intercept difference is likely caused by the dead volume of buffer present on the chip during the initial stages of the mixing process. The error bars represent one standard deviation from the mean value.
